# Role of carotid duplex in the assessment of carotid artery restenosis after endarterectomy or stenting

**DOI:** 10.3389/fneur.2023.1226220

**Published:** 2023-10-27

**Authors:** István Szegedi, Fanni Potvorszki, Zsófia Réka Mészáros, Cecilia Daniel, László Csiba, László Oláh

**Affiliations:** Department of Neurology, Faculty of Medicine, Doctoral School of Neuroscience, University of Debrecen, Debrecen, Hungary

**Keywords:** carotid artery stenting, carotid endarterectomy, carotid restenosis, ultrasound, systematic review

## Abstract

**Introduction:**

Redo carotid endarterectomy (CEA) and carotid stenting (CAS) are often performed when there is evidence of post-procedural restenosis. The incidence of restenosis after carotid reconstruction is not negligible, ranging from 5 to 33%. The diagnosis of significant internal carotid artery (ICA) restenosis is usually based on duplex ultrasound (US) criteria, mostly on peak-systolic flow velocity (PSV). However, there have been no generally accepted duplex US criteria for carotid restenosis after CAS or CEA.

**Methods:**

In this systematic review, the PubMed/ Medline and Scopus databases were screened to find trials that reported duplex US criteria for significant restenosis after CEA and/or CAS. Only those reports were analyzed in which the restenoses were also assessed by CT/MR or digital subtraction angiography as comparators for duplex US.

**Results:**

Fourteen studies met the predetermined search criteria and were included in this review. In most studies, PSV thresholds for significant in-stent ICA restenosis after CAS were higher than those for significant stenosis in non-procedurally treated (native) ICA. Many fewer studies investigated the US criteria for ICA restenosis after CEA. Despite the heterogeneous data, there is a consensus to use higher flow velocity thresholds for assessment of stenosis in stented ICA than in native ICA; however, there have been insufficient data about the flow velocity criteria for significant restenosis after CEA. Although the flow velocity thresholds for restenosis after CAS and CEA seem to be different, the large studies used the same duplex criteria to define restenosis after the two procedures. Moreover, different studies used different flow velocity thresholds to define ICA restenosis, leading to variable restenosis rates.

**Discussion:**

We conclude that (1) further examinations are warranted to determine appropriate duplex US criteria for restenosis after CAS and CEA, (2) single duplex US parameter cannot be used to reliably determine the degree of ICA restenosis, (3) inappropriate US criteria used in large studies may have led to false restenosis rates, and (4) studies are required to determine if there is a benefit from redo carotid artery procedure, such as redo-CEA or redo-CAS, starting with prospective risk stratification studies using current best practice non-invasive care alone.

## Introduction

Redo carotid endarterectomy (CEA) and carotid stenting (CAS) are often performed when there is evidence of post-procedural restenosis. The incidence of restenosis after carotid reconstruction is not negligible, ranging from 5 to 33%. The frequency of carotid restenosis after CAS and CEA depends on the definition and assessment criteria of restenosis and the duration of follow-up.

A meta-analysis of the prevalence of stroke in asymptomatic patients with severe restenosis (>70%) after CAS showed no higher risk of ipsilateral stroke than in patients without severe in-stent restenosis. However, after a mean follow-up of 37 months after CEA, the rate of ipsilateral stroke in asymptomatic patients with severe internal carotid artery (ICA) restenosis, although low (5.2%), was higher than in patients without severe restenosis (1.5%) (1). Although the treatment of carotid restenosis is highly controversial, current guidelines endorse redo CEA or CAS in a selected subgroup of symptomatic patients with restenosis between 50 and 99% (2). However, it has to be highlighted that there is no current evidence of benefit from a redo carotid artery procedure in restenosis compared to non-invasive management alone either in asymptomatic or symptomatic patients. Non-invasive care for carotid artery disease includes the identification of arterial disease risk factors and lowering arterial disease risk using healthy lifestyle practices and appropriate medication.

Carotid duplex ultrasound (US) is a popular non-invasive screening test that is used for follow-up after CEA or CAS, while computed tomography angiography (CTA) or contrast-enhanced magnetic resonance angiography (MRA) serves as an independent assessment. In most studies, restenosis rates reported after carotid artery surgery or stenting were based on duplex ultrasound findings (3–5). However, duplex ultrasound criteria for significant restenosis after CEA or CAS may differ from each other and from those used in non-procedurally treated ICA stenosis (native ICA stenosis) (6–10). Nevertheless, a number of large studies investigating restenosis rates used identical duplex US criteria to identify restenosis after CAS and CEA (3–5). Moreover, different trials applied different duplex US criteria for the definition of significant restenosis, potentially leading to false restenosis values and variable and unreliable restenosis rates after carotid stenting and carotid surgery (3–5).

In this report, we aimed to review the literature to examine the ultrasound criteria for carotid restenosis developed after CAS or CEA. Only those reports were selected, in which duplex US criteria were examined for significant restenoses estimated by CTA, MRA, or digital subtraction angiography (DSA).

## Materials and methods

A systematic review was conducted according to the recommendations of the Preferred Reporting Items for Systematic Reviews and Meta-Analyses (PRISMA) statement. The PubMed/Medline and Scopus databases were independently searched by three investigators (IS, FP and ZRM) to identify prospective or retrospective trials involving ultrasound criteria for restenosis after CEA and/or CAS. The oldest publication date of the searched articles was January 1990, while the date of the last search was 1 May 2023. Only those reports were selected for analysis, in which the ultrasound criteria were examined for at least a 50% ICA restenosis that was also estimated by CTA, MRA, or DSA. Due to the highly variable and insufficient data, quantitative analysis could not be performed.

The keywords for the search were the following:

- carotid endarterectomy AND
  restenosis AND
  (duplex OR Doppler OR ultrasound) AND
  (criteria OR threshold OR cut-off)
- carotid AND
  stent AND
  restenosis AND
  (duplex OR Doppler OR ultrasound) AND
  (criteria OR threshold OR cut-off)

Data retrieved from each constituent trial included the type of carotid intervention (CEA, CAS), the method for determining the degree of stenosis (European Carotid Surgery Trial /ECST/ or North American Symptomatic Carotid Endarterectomy Trial /NASCET/), the presence or absence of restenosis greater than a certain degree, and the ultrasound criteria. The ultrasound criteria contained cutoff values for peak systolic flow velocity (PSV), end-diastolic flow velocity (EDV), and internal carotid artery and common carotid artery peak systolic flow velocity ratio (ICA/CCA PSV ratio).

## Results

**Figure 1:**
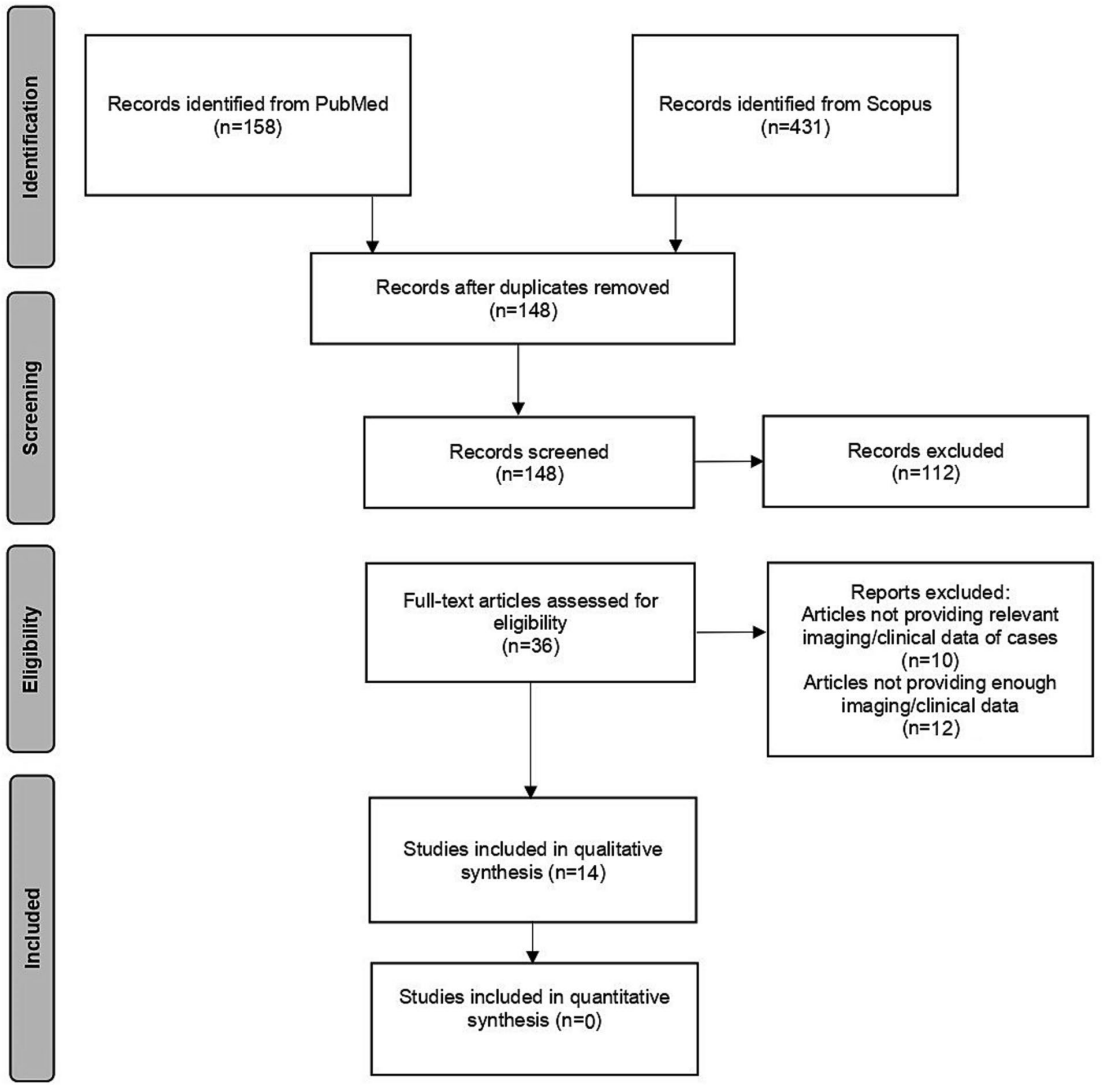
PRISMA (Preferred Reporting Items for Systematic Reviews and Meta-Analyses) flow diagram.

The literature search found 148 potentially relevant records after duplicates were removed. After screening titles and abstracts, 36 articles were selected for full-text evaluation. Fourteen studies met the predetermined search criteria and were included in this systematic review (6–9, 11–20), as shown in the PRISMA flow diagram (Figure 1). A total of 3,186 patients with previous carotid procedures were included in the studies. Tables 1–3 show the number of carotid arteries examined in each study.

**Table 1:** Duplex US criteria for moderate in-stent restenosis after CAS.

Authors	Year	No. of examined ICAs	Stenosis severity	PSV	EDV	ICA/CCA PSV ratio	Control imaging
Stanziale et al. (6)	2005	118	≥50%	225	75	2.5	DSA
Chi et al. (11)	2007	260	≥50%	240	NA	2.45	DSA
Setacci et al. (12)	2008	814	≥50%	175	NA	NA	DSA
AbuRahma et al. (13)	2008	144	≥50%	224	88	3.43	CTA/DSA
Lal et al. (7)	2008	255	≥50%	220	NA	2.7	CTA/DSA
Cumbie et al. (14)	2008	129	≥50%	195	75	2.2	DSA
Bosch et al. (9)	2017	103	≥50%	125	NA	1.5	CTA
Bitsko et al. (15)	2022	38	≥60%	240	50	2.2	CTA
Liu et al. (16)	2023	103	>50%	195	53	1.89	DSA

Stenosis severity was diagnosed by CTA, MRA, or DSA based on the NASCET criteria. CAS, carotid artery stenting; CCA, common carotid artery; CTA, computer tomography angiography; DSA, digital subtraction angiography; EDV, end-diastolic velocity; ICA, internal carotid artery; NASCET, North American Symptomatic Carotid Endarterectomy Trial; NA, not available; No., number; PSV, peak systolic velocity.

**Table 2:** Duplex US criteria for severe in-stent restenosis after CAS.

Authors	Year	No. of examined ICAs	Stenosis severity	PSV	EDV	ICA/CCA PSV ratio	Control imaging
Stanziale et al. (6)	2005	118	≥70%	350	125	4.75	DSA
Peterson et al. (17)	2005	158	≥70%	170	120	NA	DSA
Chi et al. (11)	2007	260	≥70%	450	NA	4.3	DSA
Setacci et al. (12)	2008	814	≥70%	300	140	3.8	DSA
AbuRahma et al. (13)	2008	144	≥80%	325	119	4.5	CTA/DSA
Lal et al. (7)	2008	255	≥80%	340	NA	4.15	CTA/DSA
Zhou et al. (18)	2008	282	≥70%	300	90	4	DSA
Cumbie et al. (14)	2008	129	≥80%	205	NA	2.6	DSA
Liu et al. (16)	2023	103	>80%	280	78	2.55	DSA

Stenosis severity was diagnosed by CTA, MRA, or DSA based on NASCET criteria. CAS, carotid artery stenting; CCA, common carotid artery; CTA, computer tomography angiography; DSA, digital subtraction angiography; EDV, end-diastolic velocity; ICA, internal carotid artery; NASCET, North American Symptomatic Carotid Endarterectomy Trial; NA, not available; No., number; PSV, peak systolic velocity.

**Table 3:** Duplex US criteria for restenosis after CEA.

Authors	Year	No. of examined ICAs	Stenosis severity	PSV	EDV	ICA/CCA PSV ratio	Control imaging
Telman et al. (19)	2006	268	≥70%	220	70	NA	DSA
AbuRahma et al. (8)	2009	195	≥50%	213	60	2.25	CTA/DSA
	2009		≥70%	274	80	3.35	CTA/DSA
AbuRahma et al. (20)	2011	195	≥50%	213	60	2.25	CTA/DSA
	2011		≥80%	274	94	3.35	CTA/DSA

Stenosis severity was diagnosed by CTA, MRA, or DSA based on NASCET criteria. CCA, common carotid artery; CEA, carotid artery endarterectomy; CTA, computer tomography angiography; DSA, digital subtraction angiography; EDV, end-diastolic velocity; ICA, internal carotid artery; NASCET, North American Symptomatic Carotid Endarterectomy Trial; NA, not available; No., number; PSV, peak systolic velocity.

The degree of restenosis in all studies was also estimated by DSA (*n* = 8), CTA (*n* = 2), and DSA or CTA (*n* = 4). All studies used the NASCET criteria for evaluating the degree of stenosis. There was heterogeneity in the definition of significant carotid stenosis: ≥50% or ≥ 60% stenosis was used for the definition of a less severe ICA restenosis (Tables 1, 3) and ≥ 70% or ≥ 80% for a more severe ICA restenosis (Tables 2, 3). All studies reported at least one of the PSV, EDV, and ICA/CCA ratio cutoff values for a certain degree of ICA restenosis that was also measured by CTA or DSA after CAS or CEA. MRA was not used in any of the studies included in this review.

### Duplex ultrasound results in restenosis after CAS

Eleven studies reported the flow velocity cutoff values for in-stent restenosis after CAS (6, 7, 9, 11–18). Duplex ultrasound findings were compared with the results of DSA (*n* = 7), CTA (*n* = 2), and DSA or CTA (*n* = 2). A total of 2,852 patients with previous carotid stenting were included in the studies (Tables 1, 2).

**Figure 2:**
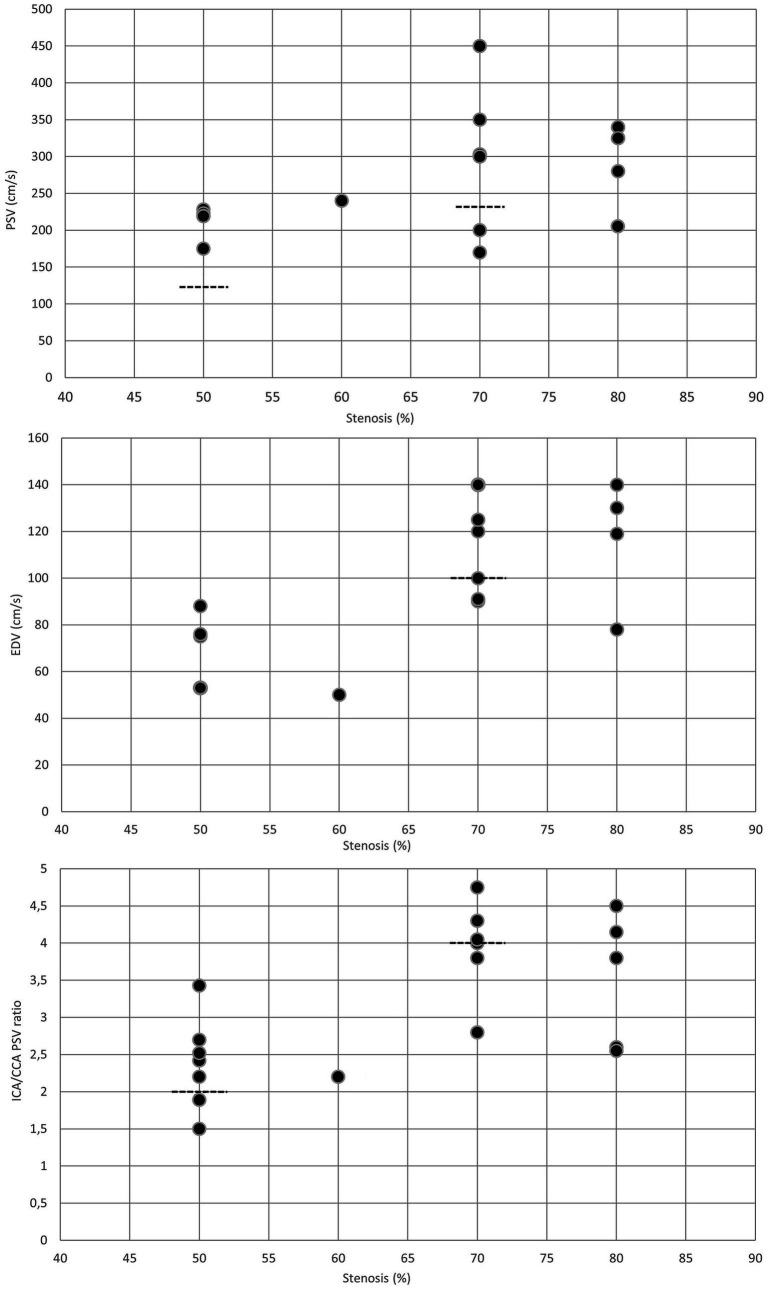
Duplex criteria for different degrees of in-stent restenosis after CAS. Dotted lines represent the standard threshold values for PSV, EDV, and ICA/CCA ratio in the corresponding graphs for ≥50% and ≥ 70% stenoses of the native internal carotid artery. CAS, carotid artery stenting; CCA, common carotid artery; EDV, end-diastolic velocity; ICA, internal carotid artery; PSV, peak systolic velocity.

For moderate (≥50%) in-stent restenosis (6, 7, 9, 11–16), PSV cutoff values varied between 125 and 240 cm/s, while EDV thresholds ranged from 50 to 88 cm/s. The ICA/CCA ratio indicating ≥50% in-stent restenosis was between 1.5 and 3.43 (Table 1 and Figure 2). Although the flow velocity thresholds for moderate in-stent restenosis showed substantial variability, most studies found that both the PSV thresholds (6, 7, 11–16) and EDV cutoff values (6, 13, 14, 16) for ≥50% in-stent restenoses after CAS were significantly higher compared to the standard PSV cutoff value for ≥50% stenosis in native ICAs. Moreover, the ICA/CCA ratio was also higher in moderate in-stent ICA restenosis (6, 7, 11, 13) than the ICA/CCA PSV ratio threshold of 2 for ≥50% native ICA stenosis (Table 1 and Figure 2).

In severe (≥70%) in-stent restenosis (6, 7, 11–14, 16–18), PSV and EDV thresholds were reported to vary between 170–450 and 78–140 cm/s, respectively. The ICA/CCA ratio for severe restenosis ranged from 2.55 to 4.75 (Table 2 and Figure 2). Similar to the moderate in-stent restenosis, the PSV cutoff values for severe in-stent restenosis were also higher (6, 11, 12, 18) than the PSV cutoff value of 230 cm/s used for classifying ≥70% stenosis in native ICA (10). EDV thresholds were also reported to be higher in severe in-stent ICA restenosis (6, 12, 17) than the standard EDV threshold of 100 cm/s for ≥70% stenosis in native ICA (10) (Table 2 and Figure 2). However, the ICA/CCA PSV ratios in severe in-stent ICA restenoses were similar (7, 11–13, 18), higher (6), or lower (14, 16) in different studies compared to the ICA/CCA PSV ratio threshold of 4 for ≥70% native ICA stenosis (Table 2 and Figure 2).

### Duplex ultrasound results in restenosis after CEA

Many fewer studies (*n* = 3) evaluated the duplex criteria for post-surgery restenosis after CEA (Table 3) (8, 19, 20) than for in-stent restenosis after CAS (*n* = 11). Moreover, AbuRahma et al. investigated the same patients in their two reports (8, 20). The degree of post-CEA restenosis was estimated by DSA in one study and by DSA or CTA in two studies. A total of 334 patients with previous carotid endarterectomy were included in the studies (Table 3).

**Figure 3:**
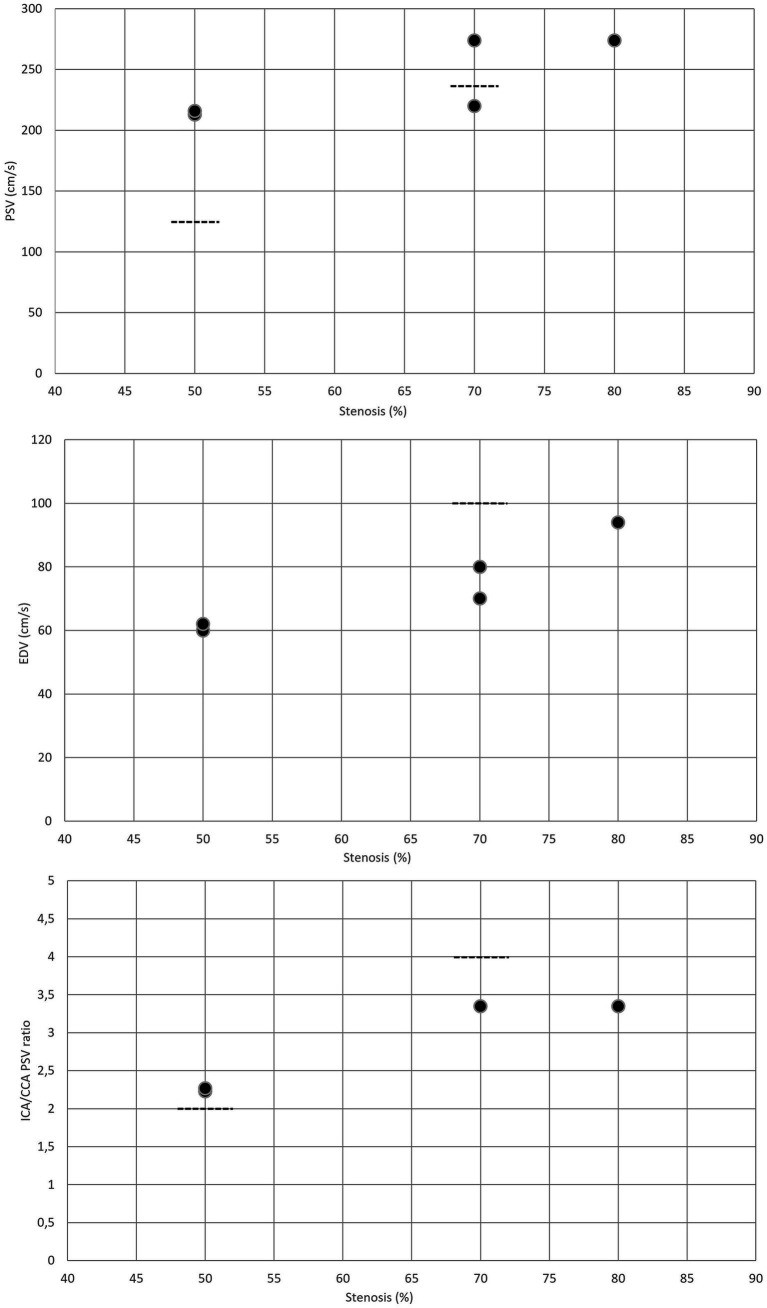
Duplex criteria for different degrees of restenosis after CEA. Dotted lines represent the standard threshold values for PSV, EDV, and ICA/CCA ratio in the corresponding graphs for ≥50% and ≥ 70% stenoses of the native internal carotid artery. CAS, carotid artery stenting; CCA, common carotid artery; EDV, end-diastolic velocity; ICA, internal carotid artery; PSV, peak systolic velocity.

For moderate (≥50%) post-surgical restenosis, the PSV cutoff value was 213 cm/s (8, 20), while the EDV threshold was 60 cm/s (8, 20). The ICA/CCA ratio indicating ≥50% restenosis after CEA was reported to be 2.25 (8, 20) (Table 3 and Figure 3). These cutoff values for ≥50% restenosis after CEA are higher than the corresponding duplex ultrasound threshold values used for classifying ≥50% stenosis in native ICA.

In severe (≥70%) post-CEA restenosis, PSV thresholds were reported to be 274 and 220 cm/s (8, 19). The EDV cutoff values indicating ≥70% restenosis were 70 and 80 cm/s, while the ICA/CCA ratio was 3.35 (Table 3 and Figure 3). These cutoff values for ≥70% post-CEA restenosis are difficult to interpret. Compared to the standard duplex ultrasound criteria for classifying ≥70% stenosis in native ICA, the PSV cutoff values reported for ≥70% post-CEA restenosis were similar or higher; however, the EDV threshold and ICA/CCA ratio were lower.

It should be emphasized, however, that due to the low number of duplex ultrasound studies investigating restenosis after CEA, no conclusions can be drawn about the ultrasound criteria for classifying post-surgical restenosis.

## Discussion

As our aim was to investigate the duplex US criteria for significant carotid restenosis, we analyzed those duplex US studies that used an independent imaging modality including DSA (*n* = 8), CTA (*n* = 2), and DSA or CTA (*n* = 4) to assess the severity of carotid restenosis and to serve as a comparator for US data. In in-stent restenosis studies, the comparator method was DSA in seven, CTA in two, and DSA or CTA also in two studies. In post-CEA restenosis trials, DSA was used in one study and DSA or CTA in two studies.

Many reports confirmed that the flow velocity thresholds for ≥50% and ≥ 70% in-stent restenoses after CAS are higher than for significant ICA stenoses in native arteries. However, due to the few studies investigating the ultrasound criteria for restenosis after carotid surgery, we have insufficient data on cutoff values for significant post-CEA restenosis. The available data after CEA suggested that the flow velocity thresholds for ≥50% restenosis are also higher, while they show no consistent trends for ≥70% restenosis compared to the standard duplex criteria for native ICA. The data also showed that despite the same method (NASCET) used for evaluation, the duplex criteria for restenosis after CAS and CEA are highly variable. The high variability of flow velocity thresholds suggests that no single duplex ultrasound parameter can be used to reliably determine the degree of ICA restenosis after CAS or CEA. Similar to the assessment of the severity of stenosis in native ICA (10), combinations of different flow velocity criteria (PSV, EDV, or ICA/CCA ratio), B-mode and color imaging, and parameters influencing cerebral hemodynamics should be considered for a more accurate evaluation of the degree of restenosis after CEA and CAS (21).

### DSA methods used to estimate the degree of carotid stenosis

In the era of the first carotid endarterectomy trials, DSA was the only imaging technique to evaluate the degree of carotid stenosis. Two basic calculation methods were used to measure the percentage reduction in the luminal diameter of ICA on DSA images: the European Carotid Surgery Trial (ECST) and the North American Symptomatic Carotid Endarterectomy Trial (NASCET) methods (22, 23). Both techniques use the luminal diameter measured at the site of the most severe stenosis, which is compared to the estimated vessel diameter at the level where the residual luminal diameter is measured in the ECST method and to the plaque-free distal ICA segment in the NASCET method. The NASCET method is more widely used, and the relevant publications we found used this method too, without exception. Therefore, the differences in the method used for the measurements of the degree of restenosis did not interfere with our analysis.

It has to be mentioned, however, that the development and spread of non-invasive imaging techniques is increasingly displacing DSA. As the results of non-invasive methods are correlated with the findings of DSA to a variable degree (24), the use of different non-invasive imaging techniques makes the evaluation of carotid stenosis difficult.

### Non-invasive methods of measuring carotid stenosis

Currently, there is no internationally accepted standard for grading carotid stenosis. Previously, catheter angiography was considered the gold standard for measuring the severity of carotid stenosis, but it has been replaced by non-invasive imaging techniques in the last decades, including MRA, CTA, and duplex ultrasound. It should be noted, however, that all carotid imaging techniques including DSA have limitations, and no method has an absolute advantage over the others (24–26). Contrast-enhanced MRA (24), which is considered the most sensitive non-invasive method, is expensive, less readily available, often overestimates the degree of carotid stenosis (27), and movement artifacts may worsen the image quality. Extensive calcification and dental amalgam may reduce the accuracy of CTA. CTA is also reported to overestimate the severity of ICA stenosis (28), while DSA, due to the limited number of projections, may lead to an underestimation of the degree of asymmetric, eccentric carotid stenosis. Although the carotid duplex is an easily available and safe technique, it can be hampered by the tortuous course of the arteries and the acoustic shadow behind calcified plaques. In addition, flow velocity measurement at the site of the stenosis is affected by contralateral ICA occlusion, collateral circulation, and the length of the stenosis, leading to a considerable variation of flow velocity values at a certain degree of ICA stenosis (10).

### Duplex US criteria for in-stent restenosis after CAS

As Table 1 demonstrates, the PSV and EDV flow velocity cutoff values, as well as the ICA/CCA PSV ratio thresholds were very heterogeneous for moderate (≥50%) in-stent restenosis after CAS. The lowest PSV cutoff value was 125 cm/s (9), just the same as for native ICA, while the highest one was 240 cm/s (11, 15), regardless of whether DSA or CTA was used for comparison. PSV thresholds for severe ICA in-stent restenosis (≥70%) were also variable, ranging from 170 to 450 cm/s, when using CTA or DSA as a comparator method (Table 2). Similar to PSV cutoff values, the EDV and ICA/CCA ratio thresholds also showed high variability for either moderate or severe in-stent restenosis.

### Duplex US criteria for restenosis after carotid surgery

It has to be emphasized that fewer studies investigated the duplex US criteria for ICA restenosis after CEA than after CAS (Table 3). Searching the literature, we have found only three studies that compared the PSV criteria for significant post-CEA restenosis with the findings of other imaging modalities (8, 19, 20), including DSA (19) and CTA or DSA (8, 20). The EDV and ICA/CCA thresholds in post-CEA restenosis were only published by AbuRahma et al. in their two articles; moreover, the results of both reports were based on data from the same patients (8, 20).

Using CTA or DSA for comparison, the PSV cutoff value for ≥50% post-CEA restenosis was 213 cm/s. Surprisingly, the PSV threshold for ≥50% restenosis (213 cm/s) was barely lower than those for ≥70% restenosis after CEA (220 and 274 cm/s, respectively).

It should be highlighted that most of the studies examined patients after CEA with patch closure (8, 19, 20), and very few data are available for restenosis after eversion carotid endarterectomy (29). However, as the standard technique for CEA today is an eversion technique, further studies are needed to determine which ultrasound criteria best predict significant restenosis after eversion CEA.

### Duplex ultrasound criteria for restenosis after CAS compared to stenosis in native ICA

Although the cutoff values for significant in-stent restenosis were variable, there was a consensus to use higher flow velocity criteria for assessment of the degree of in-stent restenosis after CAS than in native ICA (2). Based on data from Lal et al. (7) and Stanziale et al. (6), the Clinical Practice Guidelines of the European Society for Vascular Surgery (ESVS) (2) reported a 220 cm/s PSV threshold for ≥50% and a 300 cm/s cutoff value for ≥70% in-stent restenosis, compared with the 125 cm/ and 230 cm/s PSV thresholds for ≥50% and ≥ 70% stenoses in native ICAs (10), respectively. The higher flow velocity values in in-stent restenosis may lead to an overestimation of the degree of restenosis when using duplex US criteria reported for native arteries (30).

Higher flow velocity in in-stent restenosis after CAS may be due to changes in the biomechanical properties of the artery, including higher stiffness of the arterial wall, rendering the stented carotid artery similar to a rigid tube. This process decreases arterial compliance, leading to a reduced alteration of the volume of the arterial segment during different phases of the pulse wave (31).

### Duplex ultrasound criteria for restenosis after CEA compared to stenosis in native ICA

As Table 3 shows, AbuRahma et al. (8, 20) found significantly higher PSV cut-off values for ≥50% restenosis after CEA (213 cm/s), compared to the corresponding threshold value for ≥50% stenosis in native ICA (125 cm/s) (10). The PSV threshold for ≥70% carotid restenosis was found to be similar or slightly higher (220 and 274 cm/s, respectively) (8, 19) than the cut-off flow velocity for ≥70% stenosis in native ICA (230 cm/s) (10). Based on these data, the Clinical Practice Guidelines of the European Society for Vascular Surgery (ESVS) suggested different duplex US criteria for moderate and severe post-CEA restenoses compared to those in native carotid arteries (2). The ESVS guideline reported a 213 cm/s PSV threshold for ≥50% and a 274 cm/s cut-off value for ≥70% post-CEA restenosis, which values are greater than the corresponding threshold values in native carotid arteries. However, due to insufficient data for the duplex US criteria of ICA restenosis after CEA, there has been no consensus on the flow velocity thresholds indicating ≥50% or ≥ 70% post-CEA restenosis.

The use of higher thresholds in post-CEA restenosis compared to native arteries was confirmed by data from Benzing et al. (29), who investigated the duplex US criteria after longitudinal arteriotomy with patch closure and after eversion CEA. They found that using the standard 125 cm/s PSV threshold, which indicates ≥50% stenosis in native arteries, overestimated the degree of post-CEA restenosis after both surgical techniques. In carotid arteries with a PSV >125 cm/s, the percentage of the stenosis measured by CTA or MRA was only 8 ± 11 and 8% ± 35% after eversion CEA and after longitudinal arteriotomy with patch closure, respectively.

Change of conformation of carotid bifurcation, neo-intimal hyperplasia, and vascular remodeling after carotid surgery are considered to be the most likely explanations for why the standard duplex US criteria used for stenosis of native ICA do not match the criteria for ICA restenosis after carotid surgery (32).

### Duplex ultrasound criteria for in-stent restenosis after CAS compared to restenosis after CEA

As flow velocities in significant ICA restenosis after both CAS and CEA are higher than in native ICA stenosis, the use of standard duplex US criteria developed for native arteries may overestimate the degree of restenosis after CAS and CEA. Moreover, PSV threshold values after CAS seem to be higher than after CEA for estimating ≥50% and ≥ 70% restenoses. This observation is in line with the results of Lucatelly et al. who monitored ICA flow velocity changes in the 1st year after CAS and CEA and found higher flow velocities in the CAS than in the CEA group (33).

### Duplex US criteria for defining restenosis after CAS and CEA in large randomized trials

Although the definition of restenosis in large randomized controlled restenosis trials is variable, moderate and severe carotid restenoses are mostly defined by at least 50 and 70% diameter reductions, respectively. The major prospective, randomized, multicenter trials comparing the safety of CEA versus CAS were the CAVATAS (Carotid and Vertebral Artery Transluminal Angioplasty Study), ICSS (International Carotid Stenting Study), SPACE (Stent-Protected Angioplasty Versus Carotid Endarterectomy), EVA-3S (Endarterectomy versus Angioplasty in Patients with Symptomatic Severe Carotid Stenosis), and CREST (Carotid Revascularization Endarterectomy versus Stenting Trial) trials (3, 4, 30, 34–40). Table 4 shows the diagnostic methods for estimating carotid stenosis in native arteries and restenosis in procedurally treated carotid arteries in these studies (3, 4, 30, 34–40). The duplex ultrasound criteria for severe restenosis after CAS and CEA published in these trials are also shown. While the stenosis in the native carotid arteries was usually estimated by angiography (DSA, MRA, or CTA) or by a combination of duplex ultrasound and CTA or MRA, the definition of restenosis after CEA or CAS was always based on duplex ultrasound alone (Table 4).

**Table 4:** Diagnosis of native ICA stenosis and carotid restenosis in major prospective, randomized, multicentre trials comparing the safety of CEA versus CAS.

Clinical trial	Number of patients	Type of stenosis	Stenosis measurement	Diagnosis of stenosis/restenosis	Definition of severe restenosis ≥70%
CAVATAS (34)	504 (16 ASS, 488 SS)	Native stenosis	CCA method	DSA, or MRA, or CTA, or US	NA
**CAVATAS (** 34 **)**	**347**	**Restenosis**	**Not published**	**US**	**After both CEA and CAS: PSV > 230 cm/s; EDV > 110 cm/s; ICA/CCA ratio > 4**
ICSS (35)	1713 SS	Native stenosis	NASCET method	DSA, or US+MRA/CTA; exceptionally US alone	NA
**ICSS (** 4 **)**	**1530**	**Restenosis**	**NASCET method**	**US**	**After both CEA and CAS: PSV > 210 cm/s; EDV > 110 cm/s; ICA/CCA ratio > 4**
SPACE (36, 37)	1214 SS	Native stenosis	ECST, or NASCET method	US or angiography, using ECST (≥70%), or NASCET ≥50%) method	NA
**SPACE (** 38 **)**	**1136**	**Restenosis**	**ECST, or NASCET method**	**US**	**Local US criteria**
EVA-3S (39)	527 SS	Native stenosis	NASCET method	DSA, or US+MRA	NA
**EVA-3S (** 30 **)**	**527**	**Restenosis**	**NASCET method**	**US**	**After CEA: PSV > 210 cm/s; After CAS: PSV > 300 cm/s**
CREST (40)	2502 (1181 ASS, 1321 SS)	Native stenosis	NASCET method	DSA, or US, or US+CTA/MRA	NA
**CREST (** 3 **)**	**2191**	**Restenosis**	**NASCET method**	**US**	**After both CEA and CAS: PSV > 300 cm/s**

Restenosis data are indicated by bold text. ASS, asymptomatic stenosis; CAS, carotid artery stenting; CAVATAS, Carotid and Vertebral Artery Transluminal Angioplasty Study; CCA, common carotid artery; CREST, Carotid Revascularization Endarterectomy versus Stenting Trial; CEA, carotid endarterectomy; CTA, computer tomography angiography; DSA, digital subtraction angiography; ECST, European Carotid Surgery Trial; EDV, end-diastolic velocity; EVA-3S, Endarterectomy versus Angioplasty in Patients with Symptomatic Severe Carotid Stenosis; ICA, internal carotid artery; ICSS, International Carotid Stenting Study; MRA, magnetic resonance angiography; NA, not available; NASCET, North American Symptomatic Carotid Endarterectomy Trial; PSV, peak systolic velocity; SPACE, Stent-Protected Angioplasty versus Carotid Endarterectomy; SS, symptomatic stenosis; US, ultrasound.

Due to the lack of consensus on duplex criteria for restenosis, the flow velocity thresholds for severe carotid artery restenosis were highly variable. While the PSV threshold for defining carotid artery restenosis of at least 70% was 210 cm/s in the ICSS study (4) and 300 cm/s in the CREST trial (3) after both CEA and CAS, this PSV threshold indicating ≥70% restenosis in the EVA-3S study was 210 cm/s after CEA and 300 cm/s after CAS (30). Furthermore, strict duplex ultrasound criteria for significant carotid artery restenosis in the CAVATAS and SPACE studies were not reported, but the use of local or standard ultrasound criteria was recommended (34, 38). These data show that the PSV cutoff value indicating ≥70% carotid artery restenosis was very variable in the different studies, making the reliable comparison of restenosis rates between the trials impossible. Moreover, the above studies, with one exception (30), ignored the observation that the flow velocity cutoff values for significant restenosis might be different after CAS and CEA. The use of identical flow velocity criteria after the two procedures may lead to false restenosis rates and incorrect conclusions about the risk of restenosis after CAS and CEA. Prospective clinical studies are needed to validate the flow velocity thresholds for severe restenosis after invasive carotid interventions and to answer whether ultrasound criteria for stenosis in the native ICA differ from those for restenosis after CEA or CAS.

### Treatment of carotid stenosis – missing studies

Currently, there is no randomized trial data, which demonstrates a benefit of a carotid procedure compared to best practice non-invasive intervention alone in symptomatic or asymptomatic patients with carotid restenosis after CAS or CEA.

In asymptomatic patients with severe in-stent restenosis receiving non-invasive treatment alone, a meta-analysis showed a very low ipsilateral stroke rate (0.8%) compared to patients without severe in-stent restenosis (2.0%) during a 50-month follow-up after primary CAS (1, 2). Patients with in-stent restenosis were recruited into the meta-analysis studies between 2001 and 2014. Moreover, the studies included in the meta-analysis did not define the nature of non-invasive treatment alone. Therefore, ipsilateral stroke rates would most likely be even lower with current non-invasive care than as represented in the meta-analysis studies. Due to the very low stroke rate, current guidelines do not recommend an invasive carotid procedure in asymptomatic patients with severe carotid restenosis after CAS (2).

Quite the contrary, a meta-analysis revealed a higher ipsilateral stroke rate (5.2%) in non-invasively treated asymptomatic patients with severe post-CEA restenosis than in those without restenosis (1.5%) during a 37-month follow-up after primary CEA (1, 2). It should be noted, however, that the 5.2% ipsilateral stroke rate in patients with severe post-CEA restenosis in a 37-month period is considered very low. Taking into account that the studies included in the meta-analysis did not focus on non-invasive care and were performed between 1998 and 2014, the non-invasive treatment received by patients in these trials can be considered suboptimal by today’s standard. The importance of non-invasive care is further supported by this meta-analysis, showing that 97% of late ipsilateral strokes after CAS and 85% after CEA occurred in patients without evidence of significant restenosis. These data highlight that stroke rate in patients with carotid restenosis could primarily be reduced by improved non-invasive care (1).

In addition to not defining the criteria for non-invasive care, another limitation of the meta-analysis was the use of inappropriate ultrasound criteria to define severe ICA restenosis after CAS and CEA in the included studies (1). Despite the ESVS guideline recommends the use of 300 cm/s PSV as the cutoff value for diagnosing ≥70% in-stent restenosis after CAS and the PSV threshold for severe in-stent restenosis is considered higher than for severe post-CEA restenosis (2), most of the meta-analysis studies used the same PSV threshold for in-stent and post-CEA restenoses, which was much lower than the recommended 300 cm/s (1). The use of a lower PSV cutoff value may have mainly resulted in an overestimation of severe carotid in-stent restenosis, which may have led to a high rate of false positive severe in-stent restenosis, explaining the higher restenosis rate after CAS (10.0%) than after CEA (5.8%). Moreover, the overestimation of carotid in-stent restenosis might have resulted in selecting patients to a severe restenosis group without having severe restenosis, which may have contributed to the very low stroke rate in patients with severe in-stent restenosis (0.8% at 50 months; 0.19%/year) compared to those with post-CEA restenosis (5.2% at 37 months; 1.69%/year).

It should also be emphasized that the higher ipsilateral stroke rate in non-invasively treated patients with severe post-CEA restenosis compared to those without significant restenosis does not justify the benefit of invasive carotid procedures because these observational studies are out of date and did not take into account the risk of periprocedural complications (stroke, death, and myocardial infarction) of redo CEA and CAS. The benefits of invasive carotid interventions can only be proven if randomized controlled trials show their superiority over the current best medical intervention alone; however, these trials are missing. Furthermore, randomized procedural trials are not indicated, and are unethical, if average annual ipsilateral stroke rates are sufficiently close to zero with non-invasive care alone.

### Treatment of carotid restenosis—risk of periprocedural complications of redo CEA or CAS

Currently, there is no evidence of the benefit of either redo CEA or CAS for severe carotid restenosis, but there is data on the significant risk of periprocedural complications in invasive carotid procedures. Although we did not perform systematic analysis, we evaluated those trials and analyses from the PubMed database which reported the early periprocedural rates after redo CEA or carotid stenting for post-CEA restenosis and included more than 100 patients (41–56). Table 5 demonstrates that both reoperative surgery (3.3–5.0%) and CAS (0.6–5.1%) carry a significant 30-day stroke or death rate (41–56), but the 30-day myocardial infarction rate is also not negligible (Table 5).

**Table 5:** 30-day or in-hospital periprocedural complication rates of redo CEA and CAS in patients with post-CEA restenosis in a series describing more than 100 patients.

Clinical trial	Number of interventions	Type of intervention	30-day mortality	30-day stroke rate	30-day stroke/death rate	30-day MI rate
AbuRahma et al. (41)	124 (27 ASS, 97 SS)	redo CEA	0%	4.8%	4.8%	0%
Fokkema et al. (42)	212 (136 ASS, 76 SS)	redo CEA	0%	3.3%	3.3%	1.9%
Tu et al. (43)#	1846§	redo CEA	1.0%	2.8%	NP	1.3%
Krafcik et al. (44)	140§	redo CEA	0.7%	5.0%	5.0%	2.1%
Texacalidis et al. (45)#	1678§	redo CEA	1.0%	2.3%	NP	1.2%
Tu et al. (43)#	1572§	CAS	0.9%	2.4%	NP	0.3%
Arhuidese et al. (46)	2645 (1714 ASS, 931 SS)	CAS	0.9%	1.6%	2.2%	1%
Texacalidis et al. (45)#	2485§	CAS	0.5%	1.7%	NP	0.9%
Fokkema et al. (42)	220 (151 ASS, 69 SS)	CAS	0%	2.7%	2.7%	1.4%
AbuRahma et al. (47)	112§	CAS	0%	0.9%	0.9%	0%
New et al. (48)	338 (218 ASS, 140 SS)	CAS	1.2%	2.8%	3.7%	0.6%
Radak et al. (49)	319 (220 ASS, 99 SS)	CAS	0.3%	1.6%	NP	NP
Kahlberg et al. (50)	158 (137 ASS, 21 SS)	CAS	0%	0.6%	0.6%	0.6%
Midy et al. (51)	249 (214 ASS, 15 SS)	CAS	0.4%	2.4%	2.8%	0%
Cuadra et al. (52)	118 (89 ASS, 29 SS)	CAS	2.6%	2.5%	5.1%	NP
Mehta et al. (53)¶	223§	CAS	0%	1.9%	1.9%	0.4%
White et al. (54)	529 (314 ASS, 188 SS)	CAS	1.7%	3.2%	4.5%	0.8%
Mousa et al. (55)	214§	CAS	0.9%	1.4%	NP	0.9%
Hynes et al. (56)	1756 (1112 ASS, 644 SS)	CAS	0.7%	3.2%	3.7%	0.9%

The mortality, stroke, stroke and death, and MI rates relate to a pooled analysis of symptomatic and asymptomatic patients in the relevant studies. #, meta-analysis; ¶, in-hospital outcome; §, both asymptomatic and symptomatic patients were included without publishing the number of different types of patients. ASS, asymptomatic stenosis; CAS, carotid artery stenting; CEA, carotid endarterectomy; MI, myocardial infarction; NP not published; SS, symptomatic stenosis.

Table 6 shows the results of studies that separately reported the 30-day stroke or death rate of CAS and redo CEA in symptomatic and asymptomatic patients with post-CEA restenosis (41, 42, 46, 51, 54, 56). Similar to the risk of carotid procedures in patients with significant native ICA stenosis (57), the 30-day stroke or death rate of invasive carotid interventions was also higher in symptomatic (2.6–5.9%) compared with asymptomatic (2.0–3.8%) patients with severe post-CEA restenosis (Table 6). However, while stenting of native ICA stenosis carries a 1.3–1.9 times higher 30-day stroke or death rate than primary CEA in asymptomatic and a 1.8–3.0 times larger rate in symptomatic patients (57), no such difference can be found between redo CEA and CAS in patients with post-surgical restenosis. Available data show that the 30-day periprocedural stroke or death rate of redo CEA (2.9–3.7%) is similar to that of CAS (2.0–3.8%) in asymptomatic and also in symptomatic (redo CEA: 3.9–5.1%; CAS: 2.6–5.9%) patients with post-CEA restenosis (Table 6).

**Table 6:** 30-day or in-hospital periprocedural stroke or death rates of redo CEA and CAS in symptomatic (SS) and asymptomatic (ASS) patients with post-CEA restenosis.

Clinical trial	Number of intervention	Type of intervention	Stroke/death rate			Stroke rate (ASS vs. SS)
			All	ASS	SS	
AbuRahma et al. (41)	124 (27 ASS, 97SS)	redo CEA	4.8%	3.7%	5.1%	NP
Fokkema et al. (42)	212 (136 ASS, 76 SS)	redo CEA	3.3%	2.9%	3.9%	NP
Fokkema et al. (42)	220 (151 ASS, 69 SS)	CAS	2.7%	2.0%	4.4%	Higher risk in SS
Arhuidese et al. (46)	2,645 (1714 ASS, 931 SS)	CAS	2.2%	2.0%	2.6%	NP
Midy et al. (51)	249 (214 ASS, 15 SS)	CAS	2.8%	2.8%	2.9%	NP
White et al. (54)	529 (341 ASS, 188 SS)	CAS	4.5%	3.8%	5.9%	Higher risk in SS
Hynes et al. (56)	1756 (112 ASS, 644 SS)	CAS	3.7%	3.2%	4.9%	NP

Stroke and death rates are shown for all and also for asymptomatic (ASS) and symptomatic (SS) patients separately. ASS, asymptomatic stenosis; CAS, carotid artery stenting; CEA, carotid endarterectomy; MI, myocardial infarction; NP not published; SS, symptomatic stenosis.

Using data from Abbott’s study for comparison (57), we found that the 30-day stroke or death rate for redo CEA in post-CEA restenosis was similar to that for primary CEA in native ICA stenosis in both asymptomatic (2.9–3.7% versus 1.4–4.6%, respectively) and symptomatic patients (3.9–5.1% versus 3.2–10.0%, respectively). However, the 30-day periprocedural stroke or death rate for CAS in post-CEA restenosis was lower than that for primary CAS in native ICA stenosis (2.6–5.9% versus 6.0–12.1%, respectively) in symptomatic patients, while these rates were comparable in asymptomatic patients (2.0–3.8% versus 2.5–5.4%, respectively) (Table 6).

As mentioned before, transfemoral carotid artery stenting (CAS) in native carotid stenosis is associated with a higher 30-day periprocedural stroke or death rate compared to CEA (39, 40, 58), which has been attributed to embolization from the aortic arch or from the carotid plaque. In 2004, a new technique called transcarotid artery revascularization (TCAR) with flow reversal in the carotid artery was developed to avoid the manipulation of the guidewire through the aortic arch and to prevent embolization from the carotid plaque (59). Although TCAR has been rapidly adopted in the US, randomized trials were not performed to compare the efficacy and safety of TCAR with CAS, CEA, or best non-invasive care in patients with native carotid stenosis or carotid restenosis (60, 61).

### Treatment of carotid restenosis—best medical intervention

There is evidence that non-invasive best medical intervention is highly effective in carotid stenosis. Moreover, optimal treatment of vascular risk factors decreases not only the stroke risk but also all arterial disease complications, including the risk of myocardial infarction and vascular death. Abbott showed that the benefit from non-invasive medical intervention alone in patients with carotid stenosis has improved over the last 4 decades, leading to a substantial decrease of average annual ipsilateral stroke rates below 1%/year (approximately 0.8%/year) in patients with advanced asymptomatic carotid stenosis (57, 62–64).

Studying symptomatic patients with carotid stenosis awaiting revascularisation in recent randomized controlled trials (EVA-3S, SPACE, ICSS, and CREST) and in medical arms of earlier randomized controlled trials (NASCET, ECST, Veteran Affairs Cooperative Study) revealed that modern non-invasive care alone halved the stroke risk compared to that of earlier studies (65). Available data also showed that urgent best non-invasive treatment alone in symptomatic patients with significant carotid stenosis (66, 67) or with intracranial arterial stenosis was associated with a dramatic decrease in stroke risk (68).

Although the “optimal medical treatment” is described in detail in the Clinical Practice Guidelines of the European Society for Vascular Surgery from 2017, none of the large restenosis studies (4, 30, 34, 38, 40) highlighted the importance or defined the criteria of non-invasive best medical intervention. It means that the significance of optimal medical treatment was probably underestimated, which might have led to suboptimal non-invasive care and a higher rate of arterial disease complications. When designing new trials to compare the efficacy of invasive carotid procedures combined with non-invasive best medical treatment versus non-invasive best medical intervention alone, this issue should be treated as a priority.

### Current guidelines for carotid artery restenosis

Invasive carotid artery procedures in severe carotid restenosis after CEA or CAS are currently not justified. However, despite the lack of evidence for the benefit of CAS or CEA compared to the best non-invasive care alone, current guidelines endorse invasive procedures for severe carotid restenosis that is defined by PSV thresholds of 274 and 300 cm/s after CEA and CAS, respectively (2). The European Society for Vascular Surgery (ESVS) guideline (2), just adopting the same treatment criteria for carotid restenosis as for native stenosis, recommends invasive carotid procedures for severe symptomatic restenosis after CAS or CEA and suggests considering invasive treatment also for severe asymptomatic restenosis after CEA. However, it should be highlighted that there are no consensus criteria for diagnosing significant restenosis after carotid procedures, and the treatment criteria are based on the results of CEA trials performed 3–4 decades ago in patients with severe native ICA stenosis diagnosed by DSA (23, 24, 69). However, both imaging techniques and the best medical treatment options have significantly changed since that time: DSA was replaced by non-invasive imaging methods, and new and highly effective non-invasive stroke prevention strategies were introduced. As the current guideline recommendations are not supported by relevant study results, completely new clinical research is required to determine the diagnostic criteria and the best treatment strategy for carotid restenosis after CAS or CEA.

### Requirements for further studies

The current priority is to measure the average annual ipsilateral stroke rate in symptomatic and asymptomatic patients with severe carotid restenosis after CEA or CAS treated with the current best non-invasive medical care alone. A subgroup of patients with a sufficiently high risk of ipsilateral stroke despite the best non-invasive medical treatment (≈3%) should be considered for future randomized trials to answer whether redo CEA or CAS provides an additional stroke risk reduction compared to the best non-invasive medical treatment alone. Therefore, routine invasive carotid procedures should be stopped in patients with ICA restenosis after CAS or CEA in order to begin observational studies with long-term follow-up (at least 3–4 years) and to stratify the risk of ipsilateral stroke in patient subgroups with different restenosis severity. Although we focused on carotid restenosis in this report, the constant and significant improvement of non-invasive treatment alone and the outdated results of randomized carotid stenosis trials performed 3–4 decades ago (23, 24, 69) urge new research approach in patients with native carotid stenosis (57).

As risk stratification of ipsilateral stroke in patients with different restenosis severity is essential, reliable diagnostic criteria for significant carotid restenosis after CAS and CEA must be clarified. Being non-invasive, harmless, and easily available, duplex US could be the first-line tool for monitoring carotid restenosis. However, the lack of consensus on flow velocity thresholds for carotid restenosis requires new diagnostic studies. The comparison of complex US data (B-mode-imaging, PSV, EDV, and IC/CCA ratio) with the degree of restenosis assessed by angiographic methods may answer which duplex US criteria predict best the ≥50% and ≥ 70% carotid restenosis after CAS and CEA and whether these duplex US criteria are different in post-CEA and in-stent restenosis.

## Conclusion

The accurate evaluation of carotid restenosis is essential to diagnose significant carotid restenosis and to determine and compare the real restenosis rates in different carotid procedure trials. Due to the lack of consensus on duplex US criteria for carotid restenosis, further examination is warranted to find complex ultrasound criteria suitable for identifying significant ICA restenosis after invasive carotid artery procedures. However, it should be highlighted that a meaningful definition of clinically significant carotid restenosis that determines the management approach to best reduce the risk of ipsilateral stroke depends on the associated risk of stroke with the current best standards of non-invasive care alone and, subsequently, any benefit from a redo carotid procedure in addition to current best non-invasive medical care.

Our manuscript demonstrates the lack of reliable data on annual ipsilateral stroke rate in patients with severe carotid restenosis treated with current best non-invasive medical care alone, which is essential to identify a subgroup of patients with high risk of ipsilateral stroke. This could open the way for new randomized trials to determine whether redo CEA or CAS provides an additional stroke risk reduction compared to the best non-invasive medical treatment alone in this subgroup. Due to the significant periprocedural complication rate of invasive carotid artery procedures in severe carotid restenosis and the low and continuously decreasing ipsilateral stroke risk with best medical intervention alone, choosing to use current best practice non-invasive care alone is recommended until there is clear evidence that adding a carotid artery procedure improves patient outcomes.

## Data availability statement

The original contributions presented in the study are included in the article/supplementary material, further inquiries can be directed to the corresponding author.

## Author contributions

IS, FP, and ZRM systematically screened the literature and extracted the data. IS, CD, and LO processed the articles. IS, LC, and LO wrote the article. All authors have read and agreed to the published version of the article.
